# Unraveling the Mechanisms of Zinc Efficiency in Crop Plants: From Lab to Field Applications

**DOI:** 10.3390/plants11020177

**Published:** 2022-01-11

**Authors:** Gokhan Hacisalihoglu

**Affiliations:** Department of Biological Sciences, Florida A&M University, Tallahassee, FL 32307, USA; gokhan.h@famu.edu

Global food security and sustainability in the time of pandemics (COVID-19) and a growing world population are important challenges that will require optimized crop productivity under the anticipated effects of climate change. Agricultural sustainability in the time of a growing world population will be one of the major challenges in the next 50 years. Zinc (Zn) is one of the most important essential mineral nutrients required for metabolic processes, so a shortage of Zn constrains crop yield and quality worldwide. Zinc efficiency and higher growth and yield when there is low Zn supply make it a promising sustainable solution for developing cultivars that are zinc efficient.

Future crop plants need to be more Zn efficient with sustainable food yields under sub-optimal Zn conditions. Therefore, there is a substantial value in biological research aimed at understanding how plants uptake and utilize Zn.

A total of 11 articles are included in this Special Issue of “*Plants*” that provide an overview of current developments and trends in the times of high-throughput genomics and phenomics data analysis. Furthermore, this Special Issue presents research findings in various experimental models and areas ranging from maize to *Medicago* (alfalfa), flax, and sorghum.

**Hacisalihoglu** [1] outlines the variety of advances that took place in plant Zn efficiency research. Furthermore, it addresses why we need to study Zn in plants and the current understanding of Zn transport, uptake, storage, Zn efficiency under sub-optimal Zn regimes, and biofortification breeding efforts especially in food crop plants

**Mallikarjuna et al.** [2] describe a comparative transcriptome analysis of Fe and Zn deficiency in maize. In their contribution, they reported low-Zn mediated changes in transcriptome and differentially expressed candidate genes that could be further used in maize breeding programs.

**Anisimov et al.** [3] have used barley plants to describe root Zn uptake and distribution patterns together with assessing soil buffer capabilities.

**Cardini et al.** [4] have used alfalfa plants to investigate molecular mechanisms of Zn transport. They highlight 12 putative Zn transporter genes as well as differential expressions of MsHMA4, MsNAS1, MsZIP2, and MsHMA4 in roots and shoots.

**Desta et al.** [5] focused on how plant available Zn is affected by landscape position as well as its association with soil pH and other soil factors in Ethiopia.

**Lozano-Gonzales et al.** [6] have used cucumber plants to investigate potential effects of silicon application under differential Zn conditions. They discuss further results related to plant stress recovery.

**Grujcic et al.** [7] have used maize plants to investigate the nitrogen (N) effect on Zn, Fe, and Se biofortification efforts. They discuss further results related to N fertilization on improving micronutrient status in maize.

**Reynolds-Marzal et al.** [8] have used wheat plants to focus on the efficiencies of Zn and Se in a two-year field experiment in Spain. They discuss further results related to Zn and Se biofortification in wheat.

**Petschinger et al.** [9] have used moss plants to focus on metal tolerance. They discuss further results related to cell shape and cell wall thickness in mosses.

**Hacisalihoglu and Armstrong** [10] have used flax and sorghum plants to determine genetic variability and diversity for seed nutritional traits. In their contribution, they discuss further results related to daily value (% DV) and identified the top 12 flax and sorghum varieties for climate change stress and future food security.

Overall, the contributions to this Special Issue topic spans the full spectrum of Zn in plants and soils, cellular mechanisms, gene expressions, and biofortification. This Special Issue is an excellent summary of current progress with future outlooks that illustrates our increased knowledge on Zn and provides the foundation for further future research on the improvement of Zn nutrition in plants.

Finally, we encourage the readers to visit the articles published in this Special Issue of “Unraveling the Mechanisms of Zn Efficiency in Crop Plants: From Lab to Field Applications”.

## Data Availability

Not applicable.
